# Enhanced U-Pb detrital zircon, Lu-Hf zircon, δ^18^O zircon, and Sm-Nd whole rock global databases

**DOI:** 10.1038/s41597-023-02902-9

**Published:** 2024-01-09

**Authors:** Stephen J. Puetz, Christopher J. Spencer, Kent C. Condie, Nick M. W. Roberts

**Affiliations:** 1 Unaffiliated, 475 Atkinson Drive, Suite 704, Honolulu, HI 96814 USA; 2 Queen’s University, Department of Geological Sciences and Geological Engineering, Kingston, Ontario K7L 3N6 Canada; 3 New Mexico Institute of Mining and Technology, Socorro, NM 87801 USA; 4 British Geological Survey, Geochronology and Tracers Facility, Keyworth, Nottingham, NG12 5GG UK

**Keywords:** Geochemistry, Stratigraphy

## Abstract

High-quality global isotopic databases provide Earth scientists with robust means for developing and testing a variety of geological hypotheses. Database design establishes the range of questions that can be addressed, and validation techniques can enhance data quality. Here, six validated global isotopic databases provide extensive records of analyses from U-Pb in detrital zircon, Lu-Hf in zircon, Sm-Nd from whole rocks, and δ^18^O in zircon. The U-Pb detrital zircon records are segregated into three independently sampled databases. Independent samples are critical for testing the replicability of results, a key requisite for gaining confidence in the validity of a hypothesis. An advantage of our updated databases is that a hypothesis developed from one of the global detrital zircon databases can be immediately tested with the other two independent detrital zircon databases to assess the replicability of results. The independent εHf(*t*) and εNd(*t*) values provide similar means of testing for replicable results. This contribution discusses database design, data limitations, and validation techniques used to ensure the data are optimal for subsequent geological investigations.

## Background & Summary

Global databases comprised of isotopic measurements from igneous and sedimentary rocks and modern sediments^1–13^ are widely used to devise and test hypotheses regarding Earth’s evolution^4,7–11,14–29^. To further enhance the ability of Earth scientists to formulate, test, and revise existing hypotheses, this work presents one new and five expanded global isotopic databases. These include three independent global U-Pb detrital zircon databases, a greatly expanded global Lu-Hf zircon database, an enhanced and expanded global δ^18^O database coupled with Lu-Hf data, and a greatly expanded global Sm-Nd whole rock database.

These databases can be used to establish reproducible and replicable results. The words “reproducible” and “replicable” are sometimes used interchangeably, but the *Committee on Reproducibility and Replicability in Science*^30^ defines **reproducibility** as obtaining consistent results using the same input data, which include computational steps, methods, code, and conditions of analysis. This definition is synonymous with computational reproducibility. The Committee then defines **replicability** as obtaining consistent results across various studies aimed at answering the same scientific question, each of which has unique, independent data using either the same or different methodologies.

To optimize the ability of independent research teams to both reproduce and replicate results, we provide six global isotopic databases as open access. This ensures that all investigators have an unrestricted opportunity to conduct tests independently, as well as analyzing the records in ways that others have not yet considered. The new global databases promote both reproducibility and replicability in multiple ways. First, open access data, such as those made available here, are requisite for determining whether results are reproducible. Second, all records in the three detrital zircon databases are mutually exclusive of records from the other two, making the databases independent, which is requisite for testing if results are replicable. Note that data independence is unrelated to proximity of other samples. Instead, it means non-duplicate entries, referring to unique samples with unique U-Pb analyses. For example, a drill core can contain multiple independent samples with the same GPS coordinates if each sample is taken at a different depth, with each zircon being unique among all other zircons in the databases. Third, combining the independently sampled detrital zircon databases will increase the number of records, which should enhance analytical significance. Fourth, the εHf(*t*) and εNd(*t*) values from the Lu-Hf and Sm-Nd databases, respectively, provide a similar means of determining replicability from independent data. Fifth, because more than 50% of the records in the δ^18^O database also contain Lu-Hf analyses, εHf(*t*) values from the δ^18^O database can be compared to εHf(*t*) and εNd(*t*) values from the Lu-Hf and Sm-Nd databases to assess replicability from this perspective.

The remainder of this work gives details about the metadata and then explains advantages and limitations for certain types of data. The work concludes by suggesting additional details to be included in future databases – contingent on the research community subsequently reporting these crucial details as a standard requirement. The eventual goal is to augment future databases so existing geological hypotheses can be tested even more rigorously. In addition, the goals outlined here align with suggestions from the *FAIR Guiding Principles for scientific data management and stewardship*^31^ for finding and reusing scientific digital assets and the aims of the Deep-time Digital Earth project^32^ for harmonizing global deep-time Earth data and sharing global geoscience knowledge.

## Methods

All databases are compilations of published data. Source data were found by searching Google Scholar using keywords such as detrital zircon, U-Pb, Lu-Hf, whole rock, Sm-Nd, and ^18^O zircon. Journal subscriptions enabled us to downloading appropriate supplementary files, which were then transformed to conform with the data structures described in the Data Records section. The final step was to import the data into one of the six primary Excel databases (DB1 through DB6). One database contains all new analyses, primarily published after 2020. The other five databases are expansions of previously published versions, with data published after the most recent compilations now included. Furthermore, the databases generally consist of expanded metadata structures, with the added details likely paving the way for new types of analyses. This section summarizes metadata improvements from prior versions, explains metadata structures, and explains the method for simulating random sampling from regionally disproportionate non-random sampling.

### Data collection

Other than the newest U-Pb detrital zircon database, which chiefly consists of data published after 2020, the five other databases here have undergone a series of expansions and/or enhancements, of which, the current databases are the latest versions. For all the databases, classifying the exact rock type is subjective and often ambiguous. Despite these limitations, assigning lithological classifications likely increases the analytical value of the databases. For the detrital zircon databases, the word “detrital” is used in a very broad sense to include detrital sediments, non-detrital sediments, weathering deposits, and mineral deposits. From these sediments and deposits, zircons were extracted with detrital-like age distributions, and often referred to as detrital in the original research, even though the sediments/deposits are arguably not strictly composed of clastic detritus in some cases.

Six primary databases (DB1 through DB6) and six derivative databases (DB7 through DB12) were designed based on three key factors: (a) an Excel limitation of 1,048,576 records per worksheet, (b) significant degradation in Excel functionality at around 500,000 records for databases DB1, DB2, and DB3, and (c) our future research plans. However, we acknowledge that others will likely prefer methods for constructing derivative databases in ways different from those we have chosen. Accordingly, we hope the examples included here spur other research teams to optimize the data for specific research objectives. Descriptions of the databases versions and enhancements follow.

### DB1: U-Pb detrital zircon database 1

This U-Pb detrital zircon database was originally published in 2019^12^ but is now completely revised after re-downloading the original published data and re-importing them into the database, and then adding details previously excluded such as laser spot diameters, lab details, U-Pb ratios and uncertainties^33^, and other key items^13^. This revised database now contains 589,910 records. Additionally, ^207^Pb/^235^U ratios and ages are recalculated using the mean ^238^U/^235^U value of 137.818, which is posited to be representative of the average crustal composition^34^. This recalculation leaves most ^207^Pb/^235^U ratios unchanged. However, some labs still measure ^207^Pb/^235^U ratios. In those cases, the recalculations ensure a consistent analytical approach for evaluating discordant U-Pb analyses^13^.

### DB2: U-Pb detrital zircon database 2

This U-Pb detrital zircon database contains 604,553 records and was originally published in 2021^13^. Here, the database remains unrevised, with the sole exception being that ^207^Pb/^235^U ratios and ages are recalculated by using the mean ^238^U/^235^U value of 137.818^34^.

### DB3: U-Pb detrital zircon database 3

The third U-Pb detrital zircon database contains 610,451 records, mostly published after 2020. Again, these records conform to the standards of previous research^13,35^, which gives the three independent detrital zircon databases identical metadata structures.

### DB4: Lu-Hf zircon database

This database was originally published in 2019^12^ and then expanded in 2021^13^. The current version is further expanded by 46,566 records, which brings the total to 210,050 records. The records include both igneous and detrital samples, which are designated within the database. Also, measured ^176^Lu/^177^Hf and ^176^Hf/^177^Hf values were published in 2019^12^ but then eliminated in 2021^13^. These values are now restored and recorded for all records in the new Lu-Hf database.

### DB5: Sm-Nd whole rock database

This whole-rock database was originally published in 2019^12^ and is now expanded to 22,019 records, nearly double the original size. The records include both igneous and detrital samples, which are designated within the database. It is important to note that many of the ages in this database are assumed and/or assigned ages – rather than isotopically measured ages. Assumed ages are generally rounded to an integer (Ma) ending in either 00 or 50, generally making them easy to identify. For this reason, temporal tests with 50-myr or 100-myr bandwidth (bin size) that utilize this database are permissible, but we discourage using a bin size < 50-myr for temporal investigations.

### DB6: δ^18^O zircon database

This database expands upon two previous types of δ^18^O databases – the first consisting of only δ^18^O measurements^8,9^ and the second coupling Lu-Hf data with δ^18^O data^7,10^. Here, we combine the two types of databases into a single encompassing version, while also adding a significant amount of new data published since the earlier versions. All 22,428 database records have δ^18^O values; however, only 13,789 records have corresponding Lu-Hf data. Additionally, references are now converted to the structure defined in the Reference table and GPS coordinates of sample locations are now included, which were not available in the previous versions. The location data makes a significant advance by allowing investigations at the spatial as well as temporal level^36^, as well as allowing for weighted bootstrap resampling accounting for temporal and spatial bias^37^.

### DB7: Concordant core databases

This is a derivative database, being a subset of records from primary detrital zircon databases (DB1, DB2, and DB3). Specifically, the records were filtered to only include highly concordant non-metamorphic cores, defined here as cores with concordance classes ≤ 3 and with the Non-Iterative Probability (NIPr) age ≥ estimated stratigraphic age^35^. The records are filtered because U-Pb analyses with concordance classes > 3 are too imprecise to pinpoint detrital zircon age peaks. After filtering, the three databases are combined into DB7, yielding a database of 987,206 records with highly concordant U-Pb analyses. Then, the DB7 samples are sorted by publication year, and segregated into three subsets: DB8 with 296,315 records published from 1992-2017, DB9 with 310,360 records published from 2018-2020, and DB10 with 380,531 records published from 2021-2023. These databases serve different functions. DB1, DB2, and DB3 are research databases that are suitable for studying discordance, testing best age models, and similar analyses. DB7 is most suited for estimating the global U-Pb detrital zircon age distribution and similar analyses, whereas the independent databases DB8, DB9, and DB10 are suitable for testing if results are replicable.

### DB8-DB10: Independent concordant core databases

DB8, DB9, and DB10 are derivative databases from detrital zircon database DB7, being segregated by publication years of 1992 to 2017,2018 to 2020, and 2021-2023 respectively. This filtering technique allows studies aimed to assessing how properties such as laser spot diameter and U-Pb uncertainties vary over time. Of course, other means of creating independent derivative databases exist, including random selection of DB7 samples. In fact, we encourage others to conduct research using different filtering methods than those we currently prefer.

### DB11: Concordant rim database

DB11 is a derivative database, also being a subset of records from primary detrital zircon databases (DB1, DB2, and DB3). Specifically, the records were filtered from concordant rims (concordant classes ≤ 3) filtered from DB1, DB2, and DB3. This detrital zircon rim database contains 10,237 records. Among its potential uses, the global age distribution from the rim database (DB11) can be compared to the global age distribution from the core database (DB7), or alternatively, combined with DB7.

### DB12: Concordant metamorphic core database

DB12 is the final derivative database, being a subset of records from primary detrital zircon databases (DB1, DB2, and DB3). It contains 13,691 analyses from highly concordant cores, which the original research interpreted as metamorphic. These are analyses from cores with concordance classes ≤ 3 and with the NIPr age < the estimated stratigraphic age.

### Sampling densities

Large compilations of published isotopic-related data are commonly referred to as global databases. However, we are always cognizant of the fact that geological research utilizes non-random samples of Earth’s surface which are subject to a variety of socioeconomic factors that result in non-uniform coverage of the available rock record, which is itself heterogeneously distributed geographically. An open question then becomes how truly global are “global” compilations of published data? This is an important question because we expect geographic variability in many of the parameters extracted from isotopic databases. Regions and rock units that are particularly heavily sampled, either because they are readily accessible or because they are central to specific geological problems, have the potential to exert a disproportionate influence on aggregate results. “Global” databases that do not account for such geographic disparities in coverage are prone to regional distortions, which can limit their utility for testing hypotheses that require truly globally representative sampling of the available rock record.

**Fig. 1:**
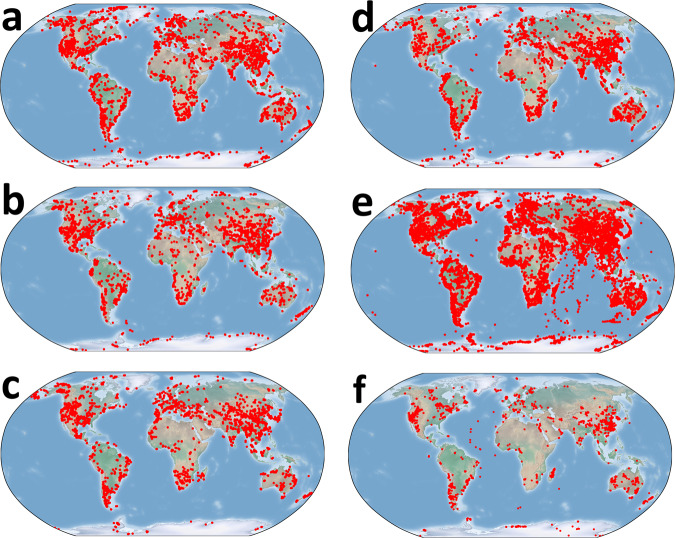
Robinson projections of samples (red dots) in the six global databases: **(a)** U-Pb detrital zircon database 1, **(b)** U-Pb detrital zircon database 2, **(c)** U-Pb detrital zircon database 3, **(d)** Lu-Hf zircon database, **(e)** Sm-Nd whole-rock database, and **(f)** δ^18^O zircon database.

By including GPS coordinates in the databases, it is possible to plot sample locations for a visual assessment of the sampling densities for all global regions, as illustrated for the six databases presented here (Fig. 1). The three detrital zircon databases (Fig. 1a–c) and the Lu-Hf zircon database (Fig. 1d) have similar disproportionate regional sampling. The Sm-Nd database (Fig. 1e) has the most geographically diverse range of samples with higher densities than the other databases. Conversely, the sampling densities are minimal for the δ^18^O database (Fig. 1f).

### Weighting samples

**Fig. 2:**
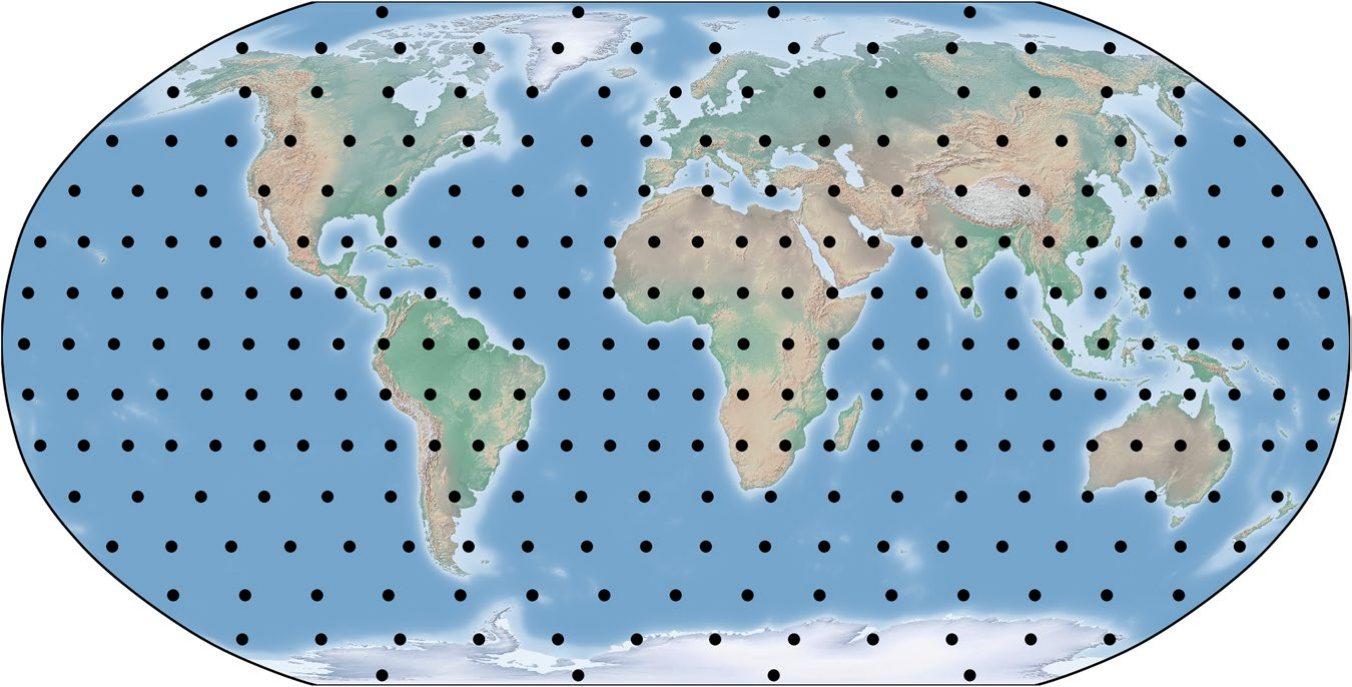
Robinson projection of centers of 12 × 12 grids (black dots) used to weight samples inversely proportional to the sampling densities illustrated in Fig. 1.

Because the samples were selected non-randomly, and because the isotopic ages and properties are heterogeneously distributed, it is imperative to weight the samples inversely proportional to the sampling densities – an approach that simulates random sampling^24,38^. The specific method used here for simulating random sampling involves a 12 × 12 grid system (Fig. 2) devised so that ~90% of the continental grids have some samples. This method is most effective when all continental regions are sampled, and its effectiveness progressively diminishes as the number of unsampled grids increases. Even while this method properly adjusts for heavily sampled grids (which are isosceles trapezoids due to Earth’s surface curvature), it is impossible to adjust for unsampled grids, unless one averages adjoining sampled grids. Here, the grids were chosen so that ~90% of them had some samples for all databases, with the δ^18^O database being the sole exception. We consider the 90% sampling threshold as more than sufficient for assessing global properties – without resorting to the more complex method of averaging adjoining grids. The database structures promote this methodology by 1) automatically calculating the grid index from the WGS84 GPS coordinates for each sample; 2) automatically tabulating the number of data records within each grid; and 3) automatically calculating the sampling weights from the grid totals.

### Segregated reference table

**Fig. 3:**
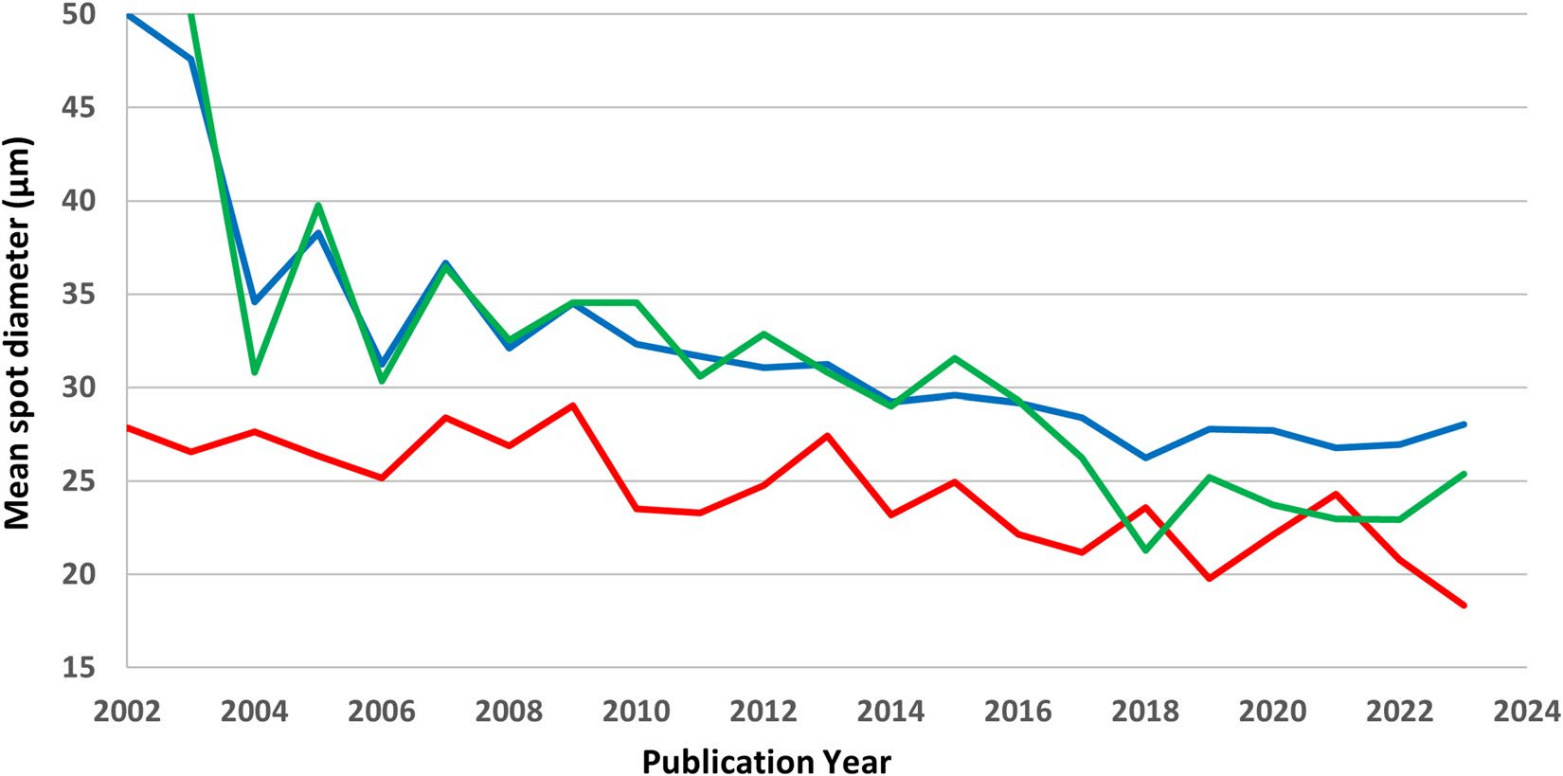
The trend toward using smaller mass spectrometer spot diameters over time for U-Pb dating of detrital zircon: (blue) LA-ICP-MS analyses for all labs, (green) LA-ICP-MS analyses conducted at the Arizona LaserChron Center, and (red) SHRIMP/SIMS analyses for all labs.

Earlier versions of some of the databases concatenated the reference details into a single reference field. Here we employ the more traditional data management technique of segregating the reference details (author names, publication year, title, journal, volume, pages, and DOI) into separate fields. The reason for applying this metadata structure might not be immediately obvious. However, two simple examples demonstrate the utility of this approach. The first example (Fig. 3) illustrates how four fields from the U-Pb detrital zircon databases – publication year, mass spectrometer type, mass spectrometer lab, and spot diameter – collectively show that spot diameters have gradually become smaller with time for all Laser Ablation Inductively Coupled Plasma Mass Spectrometry (LA-ICP-MS) labs (Fig. 3, blue), for LA-ICP-MS at the leading edge Arizona LaserChron Center lab (Fig. 3, green), and for all labs using Sensitive High Resolution Ion Microprobe (SHRIMP) and Secondary Ionization Mass Spectrometry (SIMS) methods (Fig. 3, red).

**Fig. 4:**
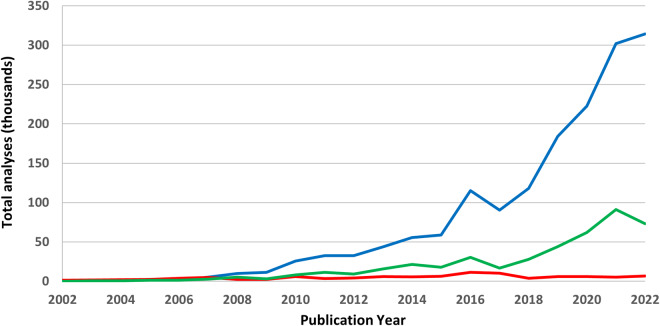
Thousands of U-Pb detrital zircon grains processed by publication year: (blue) LA-ICP-MS analyses for all labs, (green) LA-ICP-MS analyses conducted at the Arizona LaserChron Center, and (red) SHRIMP/SIMS analyses for all labs.

The second example (Fig. 4) combines information from three data fields (publication year, mass spectrometer type, and mass spectrometer lab) to demonstrate that the number of U-Pb analyses conducted annually continues to increase significantly for LA-ICP-MS analyses from all labs (Fig. 4, blue) and the single lab example presented here – the Arizona LaserChron Center (Fig. 4, green). Conversely, the annual number of U-Pb analyses from SHRIMP/SIMS methods (Fig. 4, red) has remained relatively flat over the past decade.

The usefulness of these mass spectrometry trends might be debatable, but they almost surely imply that (a) investigators increasingly use LA-ICP-MS analyses over SHRIMP/SIMS analyses, likely for both ease of access and cost efficiencies, and (b) laser spot diameters are continually being reduced to measure ever smaller zircon domains. Two decades ago, the smallest zircon domains analyzed averaged at least 50 μm in width, whereas, in 2022, the average zircon domain could be as small as 27 μm in width and still accommodate the small spot diameters. However, the exact reason for the smaller spot diameters is somewhat dubious.

Accordingly, we strive to design the metadata so that as many details as possible are recorded in the simplest, reduced formats. The role of the data administrator is to provide the data for easy access, as accurately as possible, and without judging its usefulness. When accomplished, the data analyst is then able to conduct a wider range of tests to determine which data fields add value to a specific research project.

## Data Records

The databases consist of six primary Excel spreadsheets and six derivative spreadsheets which reside in the figshare data repository^39^. Excel data are generally accessible to the entire research community, with the data easily exported to other commonly used frameworks, such as Julia^40^, Python^41^, R^42^, and MATLAB^43^. In some instances, table structures are identical for each of the six primary databases. For example, all Reference table structures are identical. Even though Excel is not a relational database, it contains functionality that mimics a primary-key/foreign-key link in a relational database. The primary-key/foreign-key link enforces referential integrity – meaning the values in certain fields are restricted to the values designated in the primary table. For databases with multiple columns with ~600,000 records, as available in the U-Pb databases, primary-key/foreign-key links in Excel cause operational speeds to degrade significantly. For this reason, in all but a few instances, links to a primary table were used once to retrieve values, and then the formulated values were converted to actual values. The time degradation only occurs when more than a million cells contain functions, equations, and other calculations. Even though the primary-key/foreign-key links are currently disabled, they can be easily restored, if required, by using the XLOOKUP function in Excel in combination with the primary and foreign keys described in the following tables. These tables briefly describe each Excel column, while also specifying its usage as either an input or calculated value. In many instances, the six databases use identical field names in similar tables. Rather than repeating descriptions, fields are listed once in the database with their initial usages, and then only fields unique to a specific database are listed thereafter. This section briefly mentions validated fields and methods, which is followed by a Technical Validation section describing more complex validations in detail.

**Reference table**. The Reference tables have identical structures, and apply to all six databases:

**Ref No**. (column A): A unique ID that serves as the primary key for the Reference table.

**Lead Author** (column B): The lead author surname followed by first name initials, with secondary authors designated with *et al*. When research includes just two authors, both surnames are given.

**Year** (column C): Year in which the research was published.

**Journal** (column D): Full or abbreviated name of the journal. To ensure referential integrity, the journal names are validated by a link to a Journal table during data acquisition.

**Vol**. (column E): Volume of the journal.

**Pages/Article** (column F): Either the page range or the article number.

**Title** (column G): Title of the research article.

**DOI or web address** (column H): The DOI or web-address for the original research article.

**Hyperlink** (column I): Clicking on any hyperlink in this column immediately navigates the computer to the website specified by the address in column H.

**Samples table**. The Samples table primarily applies to the three U-Pb detrital zircon databases. The Lu-Hf and Sm-Nd databases also contain Samples tables with similar fields, but only include a subset of the following fields. The δ^18^O database does not include a Samples table, where sample information is contained in the δ^18^O Data table.

**Ref-Sample Key** (column A): A unique ID that serves as the primary key for the Samples table, and generally linked to the foreign keys in the associated Data tables.

**Published Sample ID** (column B): The sample identification code from the original research.

**Country/Small Region** (column C): To ensure referential integrity and prevent misspellings, the country names are validated by a link to the Countries table during data acquisition. Large countries are segregated into smaller regions. For example, China is segregated at the 100th meridian east, which delineates China-Eastern from China-Western. Likewise, the United States is segregated into four regions. The Mississippi River delineates the Eastern USA from the Western USA, and the 65th parallel north divides Northern Alaska from Southern Alaska.

**Large Regions** (column D): This data field is automatically populated from a link to the Countries table. Specifically, the entry in the “Country/Small Region” field is used as the primary key to link to the Large Region field in the Countries table. For example, Africa is divided into five large regions: Northern Africa, Southern Africa, Central Africa, Eastern Africa, and Western Africa.

**Continent** (column E): The value assigned to this data field is also automatically populated from a link to the Countries table.

**Major Geographic-Geologic Description** (column F): This is a non-validated, free format field, which is generally designated by the original authors, but inferred when the authors fail to designate a major geologic unit. Examples of major geologic units include North China Craton, Central Asian Orogenic Belt, North American Cordillera, Andes Mountains, etc.

**Minor Geologic-Geographic Unit** (column G): This is also a non-validated, free format field, as designated by the original authors. This is generally a named group, formation, or member, but sometimes designated as an undifferentiated or unnamed unit.

**Locality** (column H): This is a non-validated, free format field. The locality is either assigned from the specified locality in the original research, or when not specified, assigned to a proximal locality in Google Earth^44^ after zooming toward the sample’s WGS84 GPS coordinates.

**Latitude** (column I): Regardless of the mapping system/units specified in the original research, which can be a map, UTM easting-northing, degrees-minutes-seconds, or a country-specific coordinate system, all latitudes are converted to a 5-decimal number, with minus signs designating southern hemisphere latitudes.

**Longitude** (column J): Similar to latitudes, all longitudes are converted to a 5-decimal number, with minus signs designating western hemisphere longitudes.

**Max. Stratigraphic Age (Ma)** (column K): This research abandons the term “depositional age” and now refers to this and the successive two fields as “stratigraphic ages.” When the sample is a sedimentary rock, it refers to the lithification age – which must be younger than the smallest concordant U-Pb age from the sample. Additionally, the ages of underlying intrusions and strata, as well as biomarkers and paleontological evidence, sometimes further constrain the maximum stratigraphic age to a value well below the deposition age defined by the youngest group of U-Pb ages. For modern sediments, samples are assigned a stratigraphic age of zero. For these reasons, our stratigraphic ages seldom equal the published depositional ages–although each published maximum depositional age is generally very similar to our maximum stratigraphic age, with the stratigraphic age generally being 2-myr to 20-myr younger than the maximum depositional age. As a final word of caution, interpreting depositional and stratigraphic ages often involves significant uncertainty, which gradually increases with age.

**Est. Stratigraphic Age (Ma)** (column L): The estimated stratigraphic age is the midpoint between the maximum and minimum stratigraphic ages.

**Min. Stratigraphic Age** (Ma) (column M): Like the maximum stratigraphic age, the ages of overlying intrusions and strata, as well as biomarkers and paleontological evidence, help constrain the minimum stratigraphic age – which for Precambrian samples can sometimes be significantly younger than the maximum stratigraphic age.

**Mineral** (column N): In all records, the analyzed mineral is zircon. Some of the previous versions included other minerals, which are now eliminated.

**Mass Spectrometer** (column O): This is a validated field that contains the type of mass spectrometry method used to analyze the sampled zircon grains. Valid entries are ID_TIMS, LA_ICP_MS, SHRIMP, and SIMS.

**Spectrometer Location** (column P): The primary key used to link to the Spectrometer table, to ensure referential integrity.

**Institution** (column Q): This field is automatically populated from values in the Spectrometer table – with the name of the institution and lab that performed the U-Pb analyses.

**Spectrometer Model** (column R): This field is also automatically populated from values in the Spectrometer table – with the spectrometer manufacturer and model used to perform the U-Pb analyses.

**Class-1 Rock Type** (column S): The broadest lithological classification of the sample, which is validated and constrained to be either igneous or detrital.

**Class-2 Rock Type** (column T): An intermediate lithological classification of the sample. If the Class-1 rock type is detrital, then the class-2 rock type is constrained to carbonate, siliciclastic, meta_sedimentary, modern_sediment, glacial, or misc_sediments. If the Class-1 rock type is igneous, then the class-2 rock type is constrained to a value of extrusive, intrusive, or meta_igneous.

**Class-3 Rock Type** (column U): The narrowest lithological classification of the sample. For instance, if the class-2 rock type is siliciclastic, then possible Class-3 values include conglomerate, diamictite, litharenite, matrix, mudstone, sandstone, shale, siltstone, slate, turbidite, etc.

**Ref-Sample Key** (column V): This is a duplicate value from column A, which is the primary key of the sample.

**Accepted records** (column W): For the three U-Pb detrital zircon databases, this indicates the number of records accepted from each sample, with the typical value < 100. Only records with concordance classes of 1, 2, and 3 are included. For research with specific goals and criteria, investigators can easily set the filtering criteria to a different concordance class range or to alternative field qualifiers.

**12 × 12 grid Index** (column X): An index calculated from the sampled WGS84 GPS coordinates, which is used to count the number of records within each 12° latitude x 12° longitude grid. The grid counts are then used to weight the records inversely proportional to the sampling densities of each grid.

**12 × 12 grid wt**. (column Y): The weights assigned to records, based on the values of the grid indices.

**Separator column** (column Z): Intentionally left blank.

**U-Pb Record Count** (column AA): The number of U-Pb analyses for each sample, including both accepted and rejected records. This number is generally between 4 and 600, with ~100 analyses per sample being the most common value.

**Total records** (column AB): Total number of U-Pb records in the database, including both accepted and rejected records.

**Accepted records** (column AC): Total number of accepted records in the database, using the concordance class filters set in columns AG and AH.

**% accepted** (column AD): A header that refers to the percentage at the top of column AE.

**Calculated value** (column AE): The percentage of records accepted using the filtering criteria in columns AG and AH. When the concordance range is set to 1 through 3, then approximately 50% to 60% of the records are accepted.

**Acceptance range** (classes): (column AF): Refers to inputs in columns AG and AH

**Minimum concordance class filter** (column AG):1 (accept records with concord class >  = 1)

**Maximum concordance class filter** (column AH):3 (accept records with concord class <  = 3)

**U-Pb data table**. This table only applies to the three U-Pb detrital zircon databases.

**Ref-Sample Key** (column A): The foreign key used to link to data in the Samples table.

**Sample & Grain** (column B): The sample ID and zircon analysis ID, as given in the original research.

**Zircon spot** (column C): The broadest classification of the zircon zone analyzed. Values are validated and limited to either core or rim. Cores and rims with ages less than the estimated stratigraphic age are generally interpreted as being metamorphic. Although, that might not be the case when the age is between the estimated and minimum stratigraphic ages.

**Spot diam. (μm)** (column D): The laser spot diameter, which is often an estimate rather than the exact diameter. When the original research indicates that all spot diameters were within a certain range, we assign the mean value of the range to the associated records. When analyses involve elliptical spots, we assign the mean value of the major and minor axes to the associated records. And when the original research refers to other research for analytical details, we assign the spot diameter designated in the reference to the associated records. Even while these types of estimates are less than ideal for studying records individually, they remain useful for estimating annual averages, as illustrated in Fig. 3.

**Separator column** (column E): Intentionally left blank.

^**206**^**Pb/**^**238**^**U ratio** (column F): The validated ^206^Pb/^238^U ratio, which often equals the published ^206^Pb/^238^U ratio, but not always. The validation methods for all U-Pb ratios, ages, and uncertainties are explained in the Section 4 technical validation notes.

^**206**^**Pb/**^**238**^**U 1σ uncertainty** (column G): The validated ^206^Pb/^238^U 1σ uncertainty (precision error), which is often published as absolute 2σ, a 2σ%, or a 1σ%. These are converted to 1σ uncertainties to achieve a consistent standard throughout the databases.

**Calculated** ^**207**^**Pb/**^**235**^**U ratio** (column H): The recalculated ^207^Pb/^235^U ratio, based on the mean ^238^U/^235^U value of 137.818 determined to be representative of the average crustal composition (Hiess *et al*.^34^).

^**207**^**Pb/**^**235**^**U 1σ uncertainty** (column I): The validated ^207^Pb/^235^U uncertainty (precision error), converted to 1σ.

^**207**^**Pb/**^**206**^**Pb ratio** (column J): The validated ^207^Pb/^206^Pb ratio, further explained in the Section 4 technical validation notes.

^**207**^**Pb/**^**206**^**Pb 1σ uncertainty** (column K): The validated ^207^Pb/^206^Pb uncertainty (precision error), converted to 1σ.

**Rho (published)** (column L): The published error correlation, Rho, which is calculated as either a ^207^Pb/^235^U-^206^Pb/^238^U error correlation or a ^238^U/^206^Pb-^207^Pb/^206^Pb error correlation^33^.

**Rho (calculated)** (column M): Rho, is recalculated using a ^207^Pb/^235^U-^206^Pb/^238^U error correlation, which is the method used by the Arizona LaserChron Center.

^**206**^**Pb/**^**238**^**U age (Ma)** (column N): The published/validated ^206^Pb/^238^U age, further explained in the Section 4 technical validation notes.

^**206**^**Pb/**^**238**^**U 2σ uncertainty** (column O): The published/validated ^207^Pb/^235^U uncertainty (precision error), converted to 2σ.

**Calculated** ^**207**^**Pb/**^**235**^**U age (Ma)** (column P): The recalculated ^207^Pb/^235^U age, based on the mean ^238^U/^235^U value of 137.818 determined to be representative of the average crustal composition^34^.

^**207**^**Pb/**^**235**^**U 2σ uncertainty** (column Q): The published/validated ^207^Pb/^235^U age uncertainty (precision error), converted to 2σ.

^**207**^**Pb/**^**206**^**Pb age (Ma)** (column R): The published/validated ^207^Pb/^206^Pb age, further explained in the Section 4 technical validation notes.

^**207**^**Pb/**^**206**^**Pb 2σ uncertainty** (column S): The published/validated ^207^Pb/^206^Pb age uncertainty (precision error), converted to 2σ.

**Separator column** (column T): Intentionally left blank.

**Calc** ^**206**^**Pb/**^**238**^**U age (Ma)** (column U): The ^206^Pb/^238^U age calculated directly from the published ^206^Pb/^238^U ratio, which is used to determine the validity of the published ^206^Pb/^238^U age.

**Calc** ^**206**^**Pb/**^**238**^**U 2σ uncertainty** (column V): The validated ^206^Pb/^238^U uncertainty (precision error), converted to 2σ.

**Calc** ^**207**^**Pb/**^**235**^**U age (Ma)** (column W): The ^207^Pb/^235^U age derived from the recalculated ^207^Pb/^235^U ratio, which is then used to determine the validity of the published ^207^Pb/^235^U age.

**Calc** ^**207**^**Pb/**^**235**^**U 2σ uncertainty** (column X): The validated ^207^Pb/^235^U age uncertainty (precision error), converted to 2σ.

**Calc** ^**207**^**Pb/**^**206**^**Pb age (Ma)** (column Y): The ^207^Pb/^206^Pb age calculated directly from the published ^207^Pb/^206^Pb ratio, which is used to determine the validity of the published ^207^Pb/^206^Pb age.

**Calc** ^**207**^**Pb/**^**206**^**Pb 2σ uncertainty** (column Z): The validated ^207^Pb/^206^Pb uncertainty (precision error), converted to 2σ.

**Separator column** (column AA): Intentionally left blank.

**Probability of** ^**207**^**Pb/**^**206**^**Pb age being correct** (column AB): The probability of the ^207^Pb/^206^Pb age being correct versus the ^206^Pb/^238^U age^13^.

**Non-Iter. Probability age (Ma)** (column AC): The non-iterative probability age (NIPr age)^13,35^ serves as the preferred U-Pb age in these databases.

**Non iterative 2σ uncertainty** (column AD): The 2σ uncertainty calculated from the same probabilities as the ^207^Pb/^206^Pb and ^206^Pb/^238^U ages, which equal 1 when summed.

**Min. Seg. Disc**. (column AE): An alternative measure of absolute discordance^13^.

**Base Conc. Class Delineator** (column AF): A value calculated with the NIPr age^13^, which in turn determines the concordance class for each analysis.

**Concord Class** (column AG): A calculated integer between 1 and 7^13,35^ used to classify the degree of concordance for each analysis. Analyses with a concordance class of 1 are highly concordant. As the concordance class increases, the analyses become increasingly discordant.

**12 × 12 grid index** (column AH): For details, refer to the Samples table.

**12** × **12 grid weight** (column AI): For details, refer to the Samples table.

**Hf data table**. Fields that are unique to the Lu-Hf zircon database.

**Ref-Sample Key** (column A): The foreign key used to link to data in the Samples table.

**Sample-ID** (column B): The sample ID and zircon analysis ID, as given in the original research.

**U-Pb Age (Ma)** (column C): The best U-Pb age, as determined by the original research team.

^**176**^**Yb/**^**177**^**Hf sample ratio** (column D): The measured ^176^Yb/^177^Hf ratios are available for 72.5% of the records.

^**176**^**Yb/**^**177**^**Hf 2σ** (column E): The published uncertainties, converted to 2σ, are available for 47.2% of the records.

^**176**^**Lu/**^**177**^**Hf sample ratio** (column F): The measured ^176^Lu/^177^Hf ratios are available for 100% of the records.

^**176**^**Lu/**^**177**^**Hf 2σ** (column G): The published uncertainties, converted to 2σ, are available for 60.0% of the records.

^**176**^**Hf/**^**177**^**Hf sample ratio** (column H): The measured ^176^Hf/^177^Hf ratios are available for 100% of the records.

^**176**^**Hf/**^**177**^**Hf 2σ** (column I): The published uncertainties, converted to 2σ, are available for 96.0% of the records.

***f***_**Lu/Hf**_ **calc** (column J): These values are re-calculated from (*s*/*p*)-1, where *s* is the ^176^Lu/^177^Hf sample ratio and *p* is the present day ^176^Lu/^177^Hf_CHUR_ value of 0.0332^45,46^.

**εHf(t) calc** (column K): Traditional methods are used to re-calculate εHf(t) values^47,48^. The parameters are set to a ^176^Lu decay rate of 1.867 × 10^-11^ year^49^, a ^176^Hf/^177^Hf_CHUR_ value of 0.282772^45,46^, and a ^176^Lu/^177^Hf_CHUR_ value of 0.0332^45,46^. Research teams commonly use these methods and parameters^13,50–53^, although other methods are also utilized. The re-calculated values have the advantage of providing consistent methodology throughout the database. At the same time, the database structure allows investigators to calculate these values using other methods and parameters, based on individual preferences.

**εHf(t) 2σ calc** (column L): The εHf(t) 2σ uncertainty is re-calculated using the same standard methods and parameters as given in the preceding “εHf(t) calc” section.

**T**_**DM1**_ **(Ma) calc** (column M): A recalculated single-stage Hf model age relative to the depleted mantle (DM), by using a ^176^Hf/^177^Hf_DM_ value of 0.283250^45,46^ and a ^176^Lu/^177^Hf_DM_ value of 0.0384^45,46^. While T_DM1_ ages might have some use, they tend to be highly erratic for grains within the same igneous rock, and thus tend to be unreliable.

**T**_**DM2**_ **(Ma) calc** (column N): A recalculated two-stage Hf model age relative to the depleted mantle (DM), by using the parameters described in the εHf(t) and T_DM1_ fields. For a given igneous sample, T_DM2_ ages tend to have a much smaller standard deviation than T_DM1_ ages. This suggests that T_DM2_ ages are derived from a more realistic model for the evolution of magmatic host rocks than T_DM1_ ages.

**Sm-Nd data table**. Fields that are unique to the Sm-Nd whole rock database. This table also contains data previously mentioned in the Samples table. Thus, the county, region, continent, WGS84 GPS coordinates, major and minor geologic units, rock-type 1 (igneous or detrital), and rock-type 3 details are available for analyses, just not described again here.

**Ref-Sample Key** (column A): The foreign key used to link to data in the Samples table.

**Felsic or Mafic?** (column K): For igneous samples, values in the “Class-3 Rock Type” field are used to automatically set this field to either “felsic” or “mafic.” The felsic classification includes intermediate compositions, where SiO_2_ ≥ 55%.

**Sample ID** (column L): The sample ID, as given in the original research.

**Crystallization-Age or Assumed Age (Ma)** (column M): The age of the sampled rock, as determined by the original research team. This age can be a highly precise U-Pb age, an Ar/Ar age, or and assumed age. When the age (Ma unit of time) is assumed, it is often rounded to the nearest age ending in either 50 or 00. This rounding bias leads to obvious errors in accuracy, which do not prevent using the database for time-series analysis. However, it does limit the resolution of all time-based studies. Because of the rounding, the bin-size (bandwidth) for subsequent investigations should be set to 50-myr at a minimum, but a 100-myr bin-size is preferable.

**Sampled** ^**147**^**Sm/**^**144**^**Nd** (column N): The measured ^147^Sm/^144^Nd ratio.

**Sampled** ^**143**^**Nd/**^**144**^**Nd** (column O): The measured ^143^Nd/^144^Nd ratio.

**Unreliable 1-stage T**_**DM1**_ (column P): εNd(t), T_DM1_, and T_DM2_ are re-calculated using a common method^13,50–53^. The re-calculated values have the advantage of providing consistent methodology throughout the database. Additionally, the database structure allows investigators to calculate these values using other methods, based on individual preference. Even though T_DM1_ age are provided, the typical wide variation within a single igneous sample suggests T_DM1_ model ages are too unreliable to be meaningful.

**Preferred 2-stage T**_**DM2**_ (column Q): A recalculated two-stage Nd model age relative to the depleted mantle (DM), as described in the T_DM1_ field. For a given igneous sample, T_DM2_ ages tend to have a much smaller standard deviation than T_DM1_ ages. Thus, T_DM2_ ages are likely more reliable Nd model ages.

**εNd(t)** (column R): Recalculated εNd(t) values, as described in the T_DM1_ field.

**δ**^**18**^**O data table**. Fields that are unique to the δ^18^O zircon database.

**Ref Key** (column A): The foreign key used to link to data in the Reference table.

**Analysis-Name** (column B): The sample ID, as given in the original research.

**Age (Ma)** (column K): For all detrital zircon samples, this is the best U-Pb age, as determined by the original research team. For igneous zircon samples, the ages are assigned using a variety of methods, including assumed ages.

**δ18O** (column L): δ^18^O_VSMOW_ values, which measure the ratio of the stable oxygen isotopes ^18^O and ^16^O from zircon, using either the Vienna Standard Mean Ocean Water (VSMOW) or VSMOW2 standards^54^.

**2SD** (column M): The published 2 standard deviation internal precision of the δ^18^O_VSMOW_ measurements. This could be either the standard deviation or the standard error.

**Lu-Hf data:** (columns N through S): 57.3% of the records have values for sampled ^176^Lu/^177^Hf, sampled ^176^Hf/^177^Hf, εHf(t), and re-calculated T_DM1_ and T_DM2_ model ages – all identical to the data fields and methods associated with the Lu-Hf database.

**Countries table**. All six databases are linked to the Countries table to retrieve the names of large regions, continents, country surface areas, and minimum/maximum WGS84 GPS ranges:

**Country/Small Region** (column A): A unique name that serves as the primary key for the Countries table.

**Large Region** (column B): The continents, including continental shelves, are divided into 4 to 12 large regions, with each large region consisting of multiple countries.

**Continent** (column C): Each country is assigned to a continent.

**Min Lat** (column D): The minimum latitude of the country designated by the primary key. The minimum and maximum latitudes and longitudes are used to validate the WGS84 GPS coordinates for each sample.

**Max Lat** (column E): The maximum latitude of the country designated by the primary key.

**Min Long** (column F): The minimum longitude of the country designated by the primary key.

**Max Long** (column G): The maximum longitude of the country designated by the primary key.

**Country surface area (km2)** (column H): The surface area of the country designated by the primary key.

**Journals table**. The Journals table applies to all six databases:

**Journal Names** (column A): This is the only field in this table. It serves as both a unique identifier (primary key) and the full or abbreviated journal name. This table is primarily used to ensure referential integrity for journal names.

**12 × 12 grid table**. The 12 × 12 grid table applies to all six databases, with the grids corresponding to those illustrated in Fig. 2. This is a fixed, read-only table, used to calculate grid weights that are inversely proportional to sampling densities within each grid. All grids have approximately the same surface area.

**Lat. Incr**. (column A): The integer increments for latitudinal degrees for the grids. For this table, the increment is always 12°.

**Long. Incr**. (column B): The integer increments for longitudinal degrees for the grids. For this table, the increments range from 12° at the equator to 90° at the poles. The longitudinal increments are chosen so that the surface areas of the grids near the poles have approximately the same surface areas as equatorial grids.

**Lat. Center** (column C): The latitudinal center, in degrees, of a grid. Because of Earth’s curvature, rather than being squares, each grid is an isosceles trapezoid.

**Long. Center** (column D): The longitudinal center, in degrees, of an isosceles trapezoid grid.

**Min Lat**. (column E): The minimum latitude of the grid centered at the column C value.

**Max Lat**. (column F): The maximum latitude of the grid centered at the column C value.

**Min Long**. (column G): The minimum longitude of the grid centered at the column D value.

**Max. Long**. (column H): The maximum longitude of the grid centered at the column D value.

**Dec. Index** (column I): The decimal index (primary key) assigned to the grid centered at the coordinates in columns C and D.

**Surface Area (km2)** (column J): The surface area of the grid centered at the coordinates in columns C and D.

**Continent** (column K): The name of the continent in which the center of the grid resides.

**Accepted Record Count** (column L): A conditional calculation of the number of accepted records from the Data table with WGS84 GPS coordinates corresponding to the grid defined by the primary key.

**12 × 12 Grid Weight** (column M): The calculated weight adjustment, which is inversely proportion to the sampling density of the grid defined by the primary key.

**Separator column** (column N): Intentionally left blank.

**Maximum Wt**. (column O): Extremely large weight adjustments might be inappropriate for sparsely sampled grids. For this reason, a maximum limit is set for the weight adjustments. Here, the maximum limit is set to 10, but other values can be used, perhaps with the maximum weight set to a value between 5 and 25.

**Continent** (column P): Informational only, referring to columns Q and R.

**Sampled Surface Area (km2)** (column Q): A calculated statistic, which is the sum of the grid surface areas containing at least one sample.

**Accepted records only** (column R): A calculated statistic, which is the total number of records accepted for each continent.

## Technical Validation

As data were collected from published research, and then transferred into the databases, numerous validation techniques were employed to ensure the recorded data are of the highest quality. While recording data, mistakes invariable happen along all steps of a lengthy chain of events. The frequency of mistakes is often highly dependent on the degree of automation for each step of the recording process. Specifically, processes that record data manually are more prone to errors than automated processes. As it relates to the databases here, the full chain of events begins with the original researchers collecting the samples and continues with crushing the sample, measuring isotopes, employing data reduction techniques, transferring the data into a spreadsheet or test file, and publishing the results. The full chain of events concludes with our extraction of the published data and transferring it into the final database files. Database managers typically validate data using criteria that ensures each data-field contains reasonable values. Simple validation procedures are not discussed here. These include ensuring that the publication year falls within a specified range, that the journal name matches a name in the Journal table, etc. The remainder of this section describes the more complex validation techniques utilized in this work.

### GPS validation

Precise standards are not established for recording the types of data used in the six databases here. Yet, to make the databases optimal for research purposes standards must be chosen, and then rigorously enforced when transferring published data to our formats. For example, when identifying sample locations, researchers often record the location in a map, give UTM easting-northing values, or publish coordinates in degrees, minutes, and seconds. These published units must then be converted into the standard used here: decimal WGS84 GPS coordinates. To accomplish this, comparisons are made to validate coordinates in two ways: (a) the WGS84 GPS coordinates must fall within a rectangle surrounding the selected country, as defined in the Country table, and (b) a visual comparison is made between the Google Earth location of the coordinates and the location from the published map. Even with these validation steps, some deviations occur from the actual sample sites. This is especially problematic when the original research fails to provide GPS coordinates, and only gives a low-resolution map or vague verbal descriptions of the sample locations. Nonetheless, we are reasonably confident that 99% of the samples are within 10 km of the actual sites, and the remaining 1% within 100 km of the actual sites. With these caveats in mind, if exact locations are needed, then investigators should contact the original corresponding author for sampling details.

### Validating U-Pb ratios, ages, and uncertainties

As another example of non-standard reporting, isotope-related uncertainties are typically reported in one of four formats: 1σ absolute, 1σ%, 2σ absolute, or 2σ% – with 1σ and 2σ corresponding broadly to 68% and 95% confidence levels, respectively. Here all uncertainties are converted to either 1σ or 2σ absolute values, which are specified in the Excel column header. Similarly, U-Pb ratios are reported anywhere from 2 to 12 decimal positions – with 2 decimals being insufficient for validating U-Pb ages and insufficient for calculating *IsoplotR*^55^ single grain concordia ages, whereas 12 decimals far exceed the accuracy and precision of the measurements. The standard format here is 6 decimal positions, which also likely exceeds the accuracy and precision of the measurements. However, Phanerozoic ^206^Pb/^238^U ages are far more precise than Precambrian U-Pb ages (i.e., precision varies with age), and our ongoing investigations of U-Pb accuracy and precision led to settling on 6 decimals to ensure that sufficient digits are available until the results from these studies become decisive. For the same reasons, we convert U-Pb ages to 0.1 myr resolution rather than rounding the age (Ma) to the nearest integer. The U-Pb ratios and ages are validated by using the standard equations^33^. When using these equations, the calculated ages consistently agree with U-Pb ages from the Arizona LaserChron Center, with the sole exception being that our ^207^Pb/^206^Pb 2σ uncertainties are more conservatively estimated to be ~7% greater than those from the Arizona LaserChron Center. The consistent agreement with data from the Arizona LaserChron Center suggests that its recording process is highly automated, and thus very reliable. However, the same cannot always be said for all published U-Pb data. Even while the overall data quality is reasonably high from all sources, the inability to consistently reproduce results reinforces the need to validate data and then correct erroneous records when found. When a validation attempt failed, multiple reasons were identified for the failures. These include: (a) insufficient decimal positions were reported in the U-Pb ratios, corrected by calculating the ratios directly from the ages, (b) the ^206^Pb/^238^U and ^207^Pb/^235^U ratios or ages were transposed, corrected by switching the transposed columns, (c) uncorrected ratios published with corrected ages, or vice versa, corrected by calculating the ratios directly from the ages, (d) scrambled U-Pb data files likely caused by partial sorting of columns in Excel, corrected by resorting the ratios and ages in ascending order (in a few instances, we were unable to unscramble the records, and thus rejected the entire dataset), or (e) only one U-Pb ratio or one age reported, which forced us to reject the dataset.

### Consistency in Depleted Mantle models

In yet another example of inconsistent reporting, multiple depleted mantle age models (T_DM_) have been proposed for Sm-Nd and Lu-Hf systematics. Nd model ages are often used to estimate the time of differentiation of crust from the mantle. However, if a sample is a mixture of material derived from the mantle at different times, Sm-Nd systematics only give the average time that the material has been resident in the continental crust^56^. These warnings have been repeated for Hf model ages^11,20^. Since then, investigators have formulated numerous T_DM_ models. Importantly, when researchers report T_DM_ ages, it could be any of the many models. Thus, rather than using reported T_DM_ ages, we recalculate T_DM_ for 1-stage and 2-stage models so that the given age is consistent with a specific model throughout the Sm-Nd, Lu-Hf, and O databases. Equally important, we provide the data necessary for investigators to calculate T_DM_ ages using different models with different parameters. Again, our emphasis is on consistency rather than strongly favoring one model over another.

### Validating ^176^Yb/^177^Hf ratios

Records with a ^176^Yb/^177^Hf ratio greater than the corresponding ^176^Lu/^177^Hf ratio are questionable, and thus removed from the database. This occurred with less than 0.3% of the records.

### Validating ^176^Lu/^177^Hf ratios

Approximately 0.7% of the ^176^Lu/^177^Hf records had anomalous values above the delineator, D, defined as: D = 0.02-(t/300000), where *t* is the U-Pb age in myr units. These anomalous records are rejected, having values in a range from ~0.01 to 0.5, which is consistent with typical ^176^Yb/^177^Hf values^57^. Thus, the high ^176^Lu/^177^Hf values reported in the original research likely represent accidental transpositions with ^176^Yb/^177^Hf values.

### Validating ^176^Hf/^177^Hf ratios

Sample ratios for ^176^Hf/^177^Hf are considered valid if they reside between a range from 0.2795 to 0.2837. Less than 0.3% of the records fell beyond this range.

### Validating TDM2 ages

TDM2 ages younger than the associated U-Pb age are considered erroneous and rejected. Less than 0.5% of the records failed to meet this criterion.

### Validating *f*_Lu/Hf_ values

Values for *f*_Lu/Hf_ are considered valid if they reside between a range from -0.8 to -1.0. Less than 0.3% of the records fell beyond this range.

## Current and Future Usage Notes

### Current database usages

The enlarged database structures allow for analyses and interpretations that were previously difficult or impossible to ascertain. Four examples illustrate the improved capabilities. First, the mean spot diameters for LA-ICP-MS and SHRIMP analyses have become progressively smaller over the past two decades (Fig. 3). This might imply that analyzed zircon domains have become progressively smaller over time, but without further details, this assessment remains speculative. Because a preliminary study of the U-Pb age distributions from the three detrital zircon databases are nearly identical, and because the mean spot diameter is significantly larger for detrital zircon database 1 than database 3, a preliminary and tentative conclusion is that grain size bias (including larger grains while excluding smaller grains) does not significantly alter the global U-Pb age distribution. A second example shows the number of detrital zircon analyses as a function of publication year (Fig. 4). Even though we sought data from every available article published in English, we surely missed some while including very few non-English publications. Nonetheless, the annual totals are likely indicative of the global trends in analyzing detrital zircons via LA-ICP-MS and SHRIMP/SIMS methods.

**Fig. 5:**
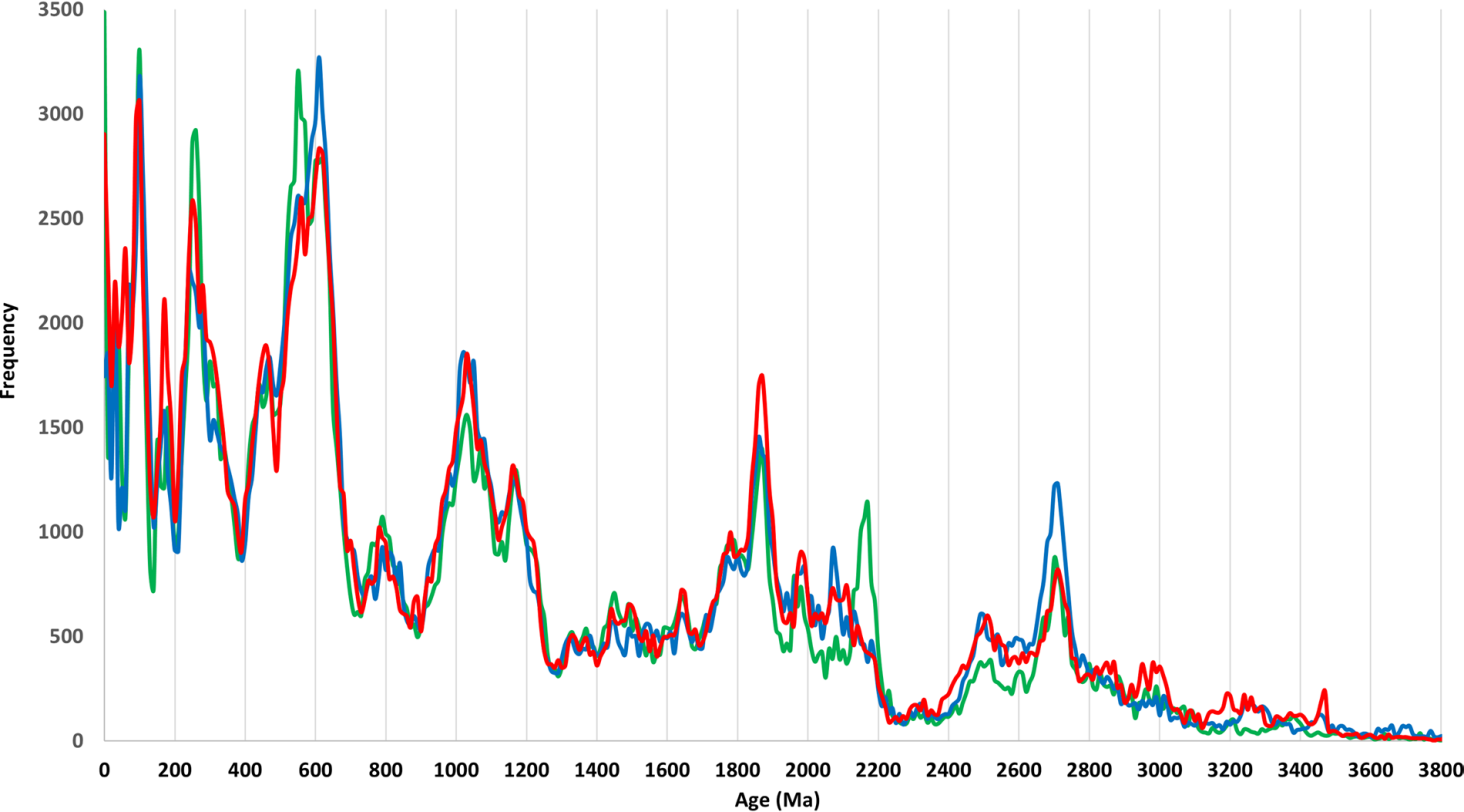
Three global U-Pb age distributions at 10-myr bandwidth (bin size): blue curve from DB8 (1992-2017 data), green curve from DB9 (2018-2020 data), and red curve from DB10 (2021-2023 data).

**Fig. 6:**
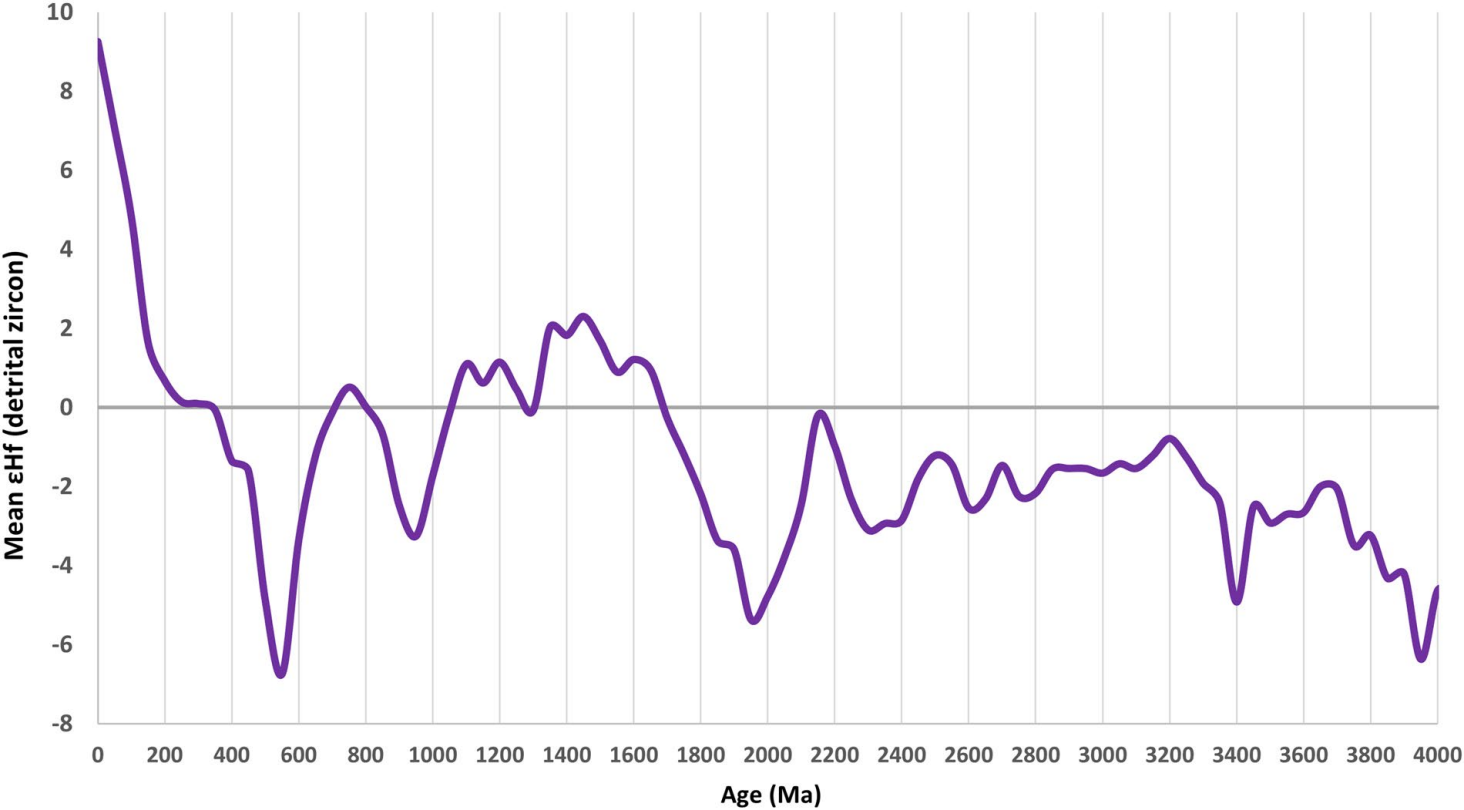
Global mean εHf(t) from detrital zircon with inverse spatial-temporal weighting.

The third and fourth examples provide high level summaries of the U-Pb and εHf(t) data. To test if global U-Pb age distributions are replicable at 10-myr resolution, frequency plots are constructed using inverse spatial weighting from the records in DB8 (Fig. 5, blue), DB9 (Fig. 5, green), and DB10 (Fig. 5, red). This methodology weights records inversely proportional to sampling densities (Fig. 1a-c). Overall, the weighted U-Pb age distributions tend to be highly replicable at age valleys, but the amplitudes of some age peaks are more variable. Lastly, a global εHf time-series (Fig. 6), constructed with inverse spatial-temporal weighting from the records in DB4, illustrates the variation in mean εHf throughout 90% of Earth’s history. Because the primary purpose of this work rests with making the data available to the research community, more detailed analyses of these data and time-series will follow in subsequent studies.

We hope these examples of database usages demonstrate that the segregated data structures in this work will pave the way for new types of global studies that were either difficult or impossible to study in the past. Along with numerous collaborators, we have already initiated several research projects to maximize usage of the records within the six primary and six secondary databases.

### Future database structures

Because of the ~1 million record limitation and functionality constraints with large record counts in Excel, converting existing databases to true relational databases will provide an optimal long-term solution for housing global geological databases. Yet, this desired goal remains elusive because of the enormous costs of developing such a system, and then maintaining dedicated staffs from both IT and geological professionals to sustain this major endeavour. Our ongoing efforts in pushing toward this direction have not yet succeeded. Thus, providing geological data in the cost-efficient Excel format remains the preferred practical alternative for sharing geological data. Specifically, supplementary Excel files are used overwhelmingly to share data, whereas text imbedded within published research is still used occasionally. Based on these database constraints and existing sharing mechanisms, periodic updates via Excel remains the only viable means for maintaining global geological databases.

We recognize that even with the enhanced structures of the databases provided here, there is always room for improvement. Some studies fail to report the desired data. In other instances, we have excluded potentially important data. Regardless of the reason, we discuss additional data fields that should enhance future research, if investigators begin reporting these items consistently.

#### Th/U ratios

Th/U is commonly used to discriminate metamorphic from magmatic zircon^58,59^, and is therefore widely used to eliminate metamorphic detrital zircon dates from datasets^60,61^. A value of 0.1 is the traditional cut-off between metamorphic (<0.1) and magmatic zircon (>0.1), despite many studies demonstrating inconsistencies with this simplistic cut-off^62,63^. It has now been demonstrated, from both empirical observations and coupled thermodynamic-accessory mineral saturation modelling that, at high temperatures, metamorphic zircon will overlap magmatic zircon in Th/U composition^64–67^. From a database of zircon analysed from Western Australia^67^, the Th/U ratios from 1352 metamorphic zircons were compared to the Th/U ratios of 5794 igneous zircons. The 25th, 50th, and 75th percentiles were 0.08, 0.44, and 1.08 for the metamorphic zircons and 0.49, 0.68, and 0.98 for the igneous zircons, respectively. From this, they^67^ conclude that zircon with Th/U ratios < 0.1 are more likely to be metamorphic and those with Th/U ratios > 0.1 can be either igneous or metamorphic.

#### Grain size values

The length, width, and depth of each zircon grain, each recorded in a separate data field, will allow rigorous testing to determine if grain size biases the global U-Pb age distribution. Currently, fewer than 1% of all published samples record this information. Furthermore, multiple investigators^68–74^ have demonstrated the value of considering grain size in various types of provenance studies. Thus, future recording of the grain size for each individual zircon grain should allow for a wider range of geologic studies.

#### Zircon grain roundness

Related to grain size, grain roundness can be calculated using an approach for sedimentary zircon^75^. One study has already done this^76^ by reporting covariation in roundness with interpreted sediment recycling. Application of automated image analysis algorithms to zircon mounts^77^ provides a means for easily including both grain size and roundness measurements along with traditional geochronological data. Including roundness measurements in large database would, for example, allow for more robust studies of the impact of detrital recycling.

#### Tectonic setting

At least since the Proterozoic, major tectonic settings for sedimentary rocks are continental arcs, oceanic arcs, collisional orogens, accretionary orogens, passive continental margins, continental rifts, cratonic basins, mid-ocean ridges (MOR), and ocean basins. For modern sands, the tectonic setting of deposition can be defined relatively unambiguously. Determining a tectonic setting for samples deposited in the geological past can be less straightforward, since it often involves a subjective assessment of geologic and geochemical data, and as with many classification systems, a clear distinction is not always possible. For Archean samples, lingering controversies about active tectonic settings obscure the approach entirely. The age spectra of grains within individual samples can be used to infer the tectonic setting via an automated process^78,79^; however, prior determinations of tectonic settings for database samples would be valuable for refining such methods, provided that circular reasoning is avoided.

### Supplementary information

DB1 - 2019 U-Pb detrital zircon database

DB2 - 2021 U-Pb detrital zircon database

DB3 - 2023 U-Pb detrital zircon database

DB4 - Yb-Lu-Hf zircon database

DB5 - Sm-Nd whole rock database

DB6 - Database of δ18O-zircon coupled with Lu-Hf

DB7 - All non-metamorphic detrital zircon class 1-3

DB8 - 1992-2017 non-metamorphic detrital zircon

DB9 - 2018-2020 non-metamorphic detrital zircon

DB10 - 2021-2023 non-metamorphic detrital zircon

DB11 - U-Pb detrital zircon rims

DB12 - U-Pb metamorphic detrital zircon cores

## Data Availability

None of the code or equations are new. All methods are from previously published research. The code used to validate the data fields are standard Excel functions.
